# 401 consecutive minimally invasive distal pancreatectomies: lessons learned from 20 years of experience

**DOI:** 10.1007/s00464-021-08997-8

**Published:** 2022-01-31

**Authors:** Alessandro Esposito, Marco Ramera, Luca Casetti, Matteo De Pastena, Martina Fontana, Isabella Frigerio, Alessandro Giardino, Roberto Girelli, Luca Landoni, Giuseppe Malleo, Giovanni Marchegiani, Salvatore Paiella, Antonio Pea, Paolo Regi, Filippo Scopelliti, Massimiliano Tuveri, Claudio Bassi, Roberto Salvia, Giovanni Butturini

**Affiliations:** 1 Department of General and Pancreatic Surgery, The Pancreas Institute, University of Verona Hospital Trust, Piazzale L.A. Scuro, 10, 37134 Verona, Italy; 2 Department of Surgery, Pederzoli Hospital, Peschiera, Italy; 3 Università di Verona, Verona, Italy

**Keywords:** Laparoscopic surgery, Robotic surgery, Distal pancreatectomy, Pancreatic ductal adenocarcinoma, Learning curve, Trocar site recurrence

## Abstract

**Background:**

This study aimed to discuss and report the trend, outcomes, and learning curve effect after minimally invasive distal pancreatectomy (MIDP) at two high-volume centres.

**Methods:**

Patients undergoing MIDP between January 1999 and December 2018 were retrospectively identified from prospectively maintained electronic databases. The entire cohort was divided into two groups constituting the “early” and “recent” phases. The learning curve effect was analyzed for laparoscopic (LDP) and robotic distal pancreatectomy (RDP). The follow-up was at least 2 years.

**Results:**

The study population included 401 consecutive patients (LDP *n* = 300, RDP *n* = 101). Twelve surgeons performed MIDP during the study period. Although patients were more carefully selected in the early phase, in terms of median age (49 vs. 55 years, *p* = 0.026), ASA class higher than 2 (3% vs. 9%, *p* = 0.018), previous abdominal surgery (10% vs. 34%, *p* < 0.001), and pancreatic adenocarcinoma (PDAC) (7% vs. 15%, *p* = 0.017), the recent phase had similar perioperative outcomes. The increase of experience in LDP was inversely associated with the operative time (240 vs 210 min, *p* < 0.001), morbidity rate (56.5% vs. 40.1%, *p* = 0.005), intra-abdominal collection (28.3% vs. 17.3%, *p* = 0.023), and length of stay (8 vs. 7 days, *p* = 0.009). Median survival in the PDAC subgroup was 53 months.

**Conclusion:**

In the setting of high-volume centres, the surgical training of MIDP is associated with acceptable rates of morbidity. The learning curve can be largely achieved by several team members, improving outcomes over time. Whenever possible resection of PDAC guarantees adequate oncological results and survival.

**Supplementary Information:**

The online version contains supplementary material available at 10.1007/s00464-021-08997-8.

In the last 20 years, minimally invasive distal pancreatectomy (MIDP) has gained popularity even if its superiority over the open technique is still debated, especially for pancreatic ductal adenocarcinoma (PDAC). In this period, several retrospective and non-randomized studies have recognised its feasibility and safety [1–8]. More recently, two randomized trials have shown that the minimally invasive approach guarantees reduced length of hospital stay (LoS) and blood loss [9, 10]. Based on this evidence, the Miami international guidelines stated that MIDP for benign and low-grade tumours should be preferred over the standard open distal pancreatectomy (OPD) approach [11]. However, MIDP diffusion is still a long process for several reasons: the complex training programs, with variations between different regions even in high-volume centres [12, 13]; the lack of robust data on oncological outcome for PDAC; the uncertainty about real advantages for patients; and the costs for the procedure itself.

Indeed, the traditional surgical teaching model adopted for open surgery is hard to apply to the minimally invasive approach. Trainees should learn by doing, rather than by observing [14]. Simulators or training platforms are useful to familiarise with the technique and memorise all steps for the procedures. However, active trainee participation during surgery is crucial for the success of a minimally invasive curriculum [15].

Several studies assessed the safety and efficacy of MIDP [16, 17]. However, even if MIDP is not as technically demanding as cephalic pancreatic resections, it remains a challenging procedure as evidenced by the high open conversion rates of 16%–31% reported even from high-volume specialised centres, especially by low experience surgeons during the learning phase [18, 19]. Some issues are still open to debate regarding indications and outcomes of MIDP: oncological results for PDAC resections; how and when to preserve the spleen; how to manage the pancreatic stump; and how to teach younger surgeons the procedure. In this study, we review the evolution and outcomes of MIDP at two high-volume institutions with over twenty years of experience and report the lessons that have been learned.

## Materials and methods

The study was performed according to the Strengthening the Reporting of Observational Studies in Epidemiology (STROBE) and Strengthening the Reporting of Cohort Studies in Surgery (STROCSS) guidelines [20, 21]**.** Patients undergoing MIDP between January 1999 and December 2018 at the General and Pancreatic Surgery Unit, Pancreas Institute, Verona University Hospital Trust, Verona, Italy, and at the Pancreatic Surgery Unit of Pederzoli Hospital in Peschiera del Garda, Verona, Italy, were retrospectively identified from prospectively maintained databases. The two institutional review boards that oversee these units approved the study, and data were obtained from the institutional patient registries. Data on demographics, pathology, operative technique, and perioperative outcomes were examined. Each patient had a follow-up of 2 years minimum.

### Surgical techniques

Each patient first underwent contrast-enhanced cross-sectional imaging of the abdomen. All cases were preoperatively reviewed at a dedicated institutional surgical meeting where the decision to perform a minimally invasive procedure was undertaken among staff surgeons [22]. Indications for a minimally invasive approach were benign or pre-malignant lesions smaller than 10 cm or, for malignancies, tumours without evidence of major vessel involvement. Decisions concerning use of a robot and the type of procedure were based on the availability of the Da Vinci Surgical System® and the surgeon's judgment. The surgeons of both centres matriculated from the same Verona University Surgical School residency program. Except for the first two surgeons who did not receive specific training or supervision, the subsequent trainees who approached MIDP were supervised by a more experienced surgeon for at least the first 30 cases. Generally, even in subsequent cases the main surgeon was assisted by a surgeon with previous experience in MIDP. Residents rotate in both hospitals but generally act as second assistant surgeons in MIDPs.

Laparoscopic distal pancreatectomy (LDP) and robotic distal pancreatectomy (RDP) were carried out as previously reported [23, 24]. For LDP, the patient is placed in supine position, 20–25° reverse Trendelenburg, 15–20° right-lilted. The first 12-mm trocar is placed above the umbilicus (camera). Then 5-mm trocar is placed in epigastrium underneath the left costal margin. The third 5-mm trocar placed in right hypochondrium, on the midclavicular line and above the transverse umbilical line. Finally, a 12-mm port is placed in left hypochondrium, lateral to the umbilicus and on the midclavicular line. An additional 5-mm port may be placed in the left hypochondrium more laterally to optimize exposure. In RDP, 5 trocars are used: four 8-mm robotic ports along a transverse umbilical line (R1, in the right flank—R2, in the right pararectal area—R3, in the periumbilical area (camera)—R4, in the left flank), and a 12-mm port assistant port underneath and between R3 and R4. A standard distal pancreatectomy with splenectomy (DP-S) and lymphadenectomy were performed for malignant lesions. Radical antegrade modular pancreatosplenectomy was performed for pancreatic body and tail malignant tumours in which posterior margin involvement was suspected. In benign neoplasms, the preservation of the spleen was always attempted. If the neoplasm was in close contact with the splenic vessels, the Warshaw technique was chosen at first. In all other cases in which preservation of splenic vessels was feasible, Kimura's method was preferred [25, 26]. Pancreatic transection The surgical field was drained by a Penrose-type surgical tube placed proximal to the pancreatic remnant. The drain was managed in the postoperative course according to the institutional protocol [27].

### Surgical Outcomes

Demographic characteristics included sex, age, body mass index (BMI, kg/m^2^), American Society of Anesthesiologists (ASA) physical status, and previous abdominal surgery. Intraoperative variables collected were: type of procedure, conversion rate, operative time (minutes), estimated blood loss (mL), and pancreatic stump management (stapler, ultrasonic scalpel, or others). Due to the retrospective nature of the study, success or failure in spleen preservation could not be analysed. Postoperative complications were recorded up to 90 days after surgery or in any case during the same hospitalisation and were graded according to the Clavien-Dindo classification, defining the major complications as Clavien-Dindo grade III or higher [28]. Postoperative pancreatic fistula (POPF), post-pancreatectomy hemorrhage (PPH), and chyle leak were defined according to the International Study Group of Pancreatic Fistula criteria [29–31]. Pathological data was also reported, including final histology, radial resection margins, and the number of lymph nodes harvested. A subgroup analysis comparing MIDP for PDAC vs. other indications was carried out.

### Time trends and learning curve

The surgical caseload was represented as a case-sequence number, and the study period was divided into two groups. The first phase described the “early” experience and included the first 201 cases performed from 1999 to 2014. During this phase, the efforts of two surgeons focused on acquisition of skills, standardisation of the technique, and initial surgical training of other team members. Furthermore, an accurate and thoughtful patient selection was performed to guarantee safety during the surgical training. The following “recent” phase included the last 200 cases from 2015 to 2018 and all team members utilised the MIDP approach.

The learning curve was defined as achieved based upon cut-offs reported in the literature, namely 17 LDP and 10 RDP procedures [32, 33]. The learning curve effect was analyzed for LDP and RDP by comparing all operator cases before and after the threshold.

### Follow-up information

The follow-up included a detailed clinical examination, blood tests (including glycemia and/or glycate hemoglobin), and cross-sectional imaging or transabdominal ultrasound, as appropriate. It was performed on a 6-month basis for the first 1–2 years and yearly after that for up to 5 years. Patients affected by PDAC received a closer surveillance, every 3 months, during the first 2 years and extending to 6 months thereafter. During the follow-up, a meticulous examination of patients was performed, particularly the trocar sites.

Diabetes mellitus (DM) was diagnosed according to the American Diabetes Association criteria [34]. For subgroup analysis of new-onset type 3c diabetes mellitus (NODM) incidence, previously diagnosed diabetic patients were excluded.

In patients who underwent splenectomy, data on compliance to post-splenectomy vaccine schedule and persistent secondary thrombocytosis (defined as a platelet count greater than 400,000/μL for at least one year after splenectomy) were collected. Of note, patients who initially underwent a spleen-preserving distal pancreatectomy (SPDP) but were splenectomised during reoperation were included in the DP-S group for this specific analysis.

### Statistical analysis

Continuous variables were expressed as median with interquartile range (IQR). The Mann–Whitney *U* test was used to compare distribution of the two groups. The χ^2^ test (with Yates continuity correction in a 2 × 2 contingency table) was used for nominal data. The Fisher’s exact test was used when appropriate. All tests were 2-tailed. A *p* value < 0.05 was considered significant. The statistical analysis was performed using SPSS software, release 24 (SPSS Inc., Chicago, IL).

## Results

### Patient selection

**Fig. 1:**
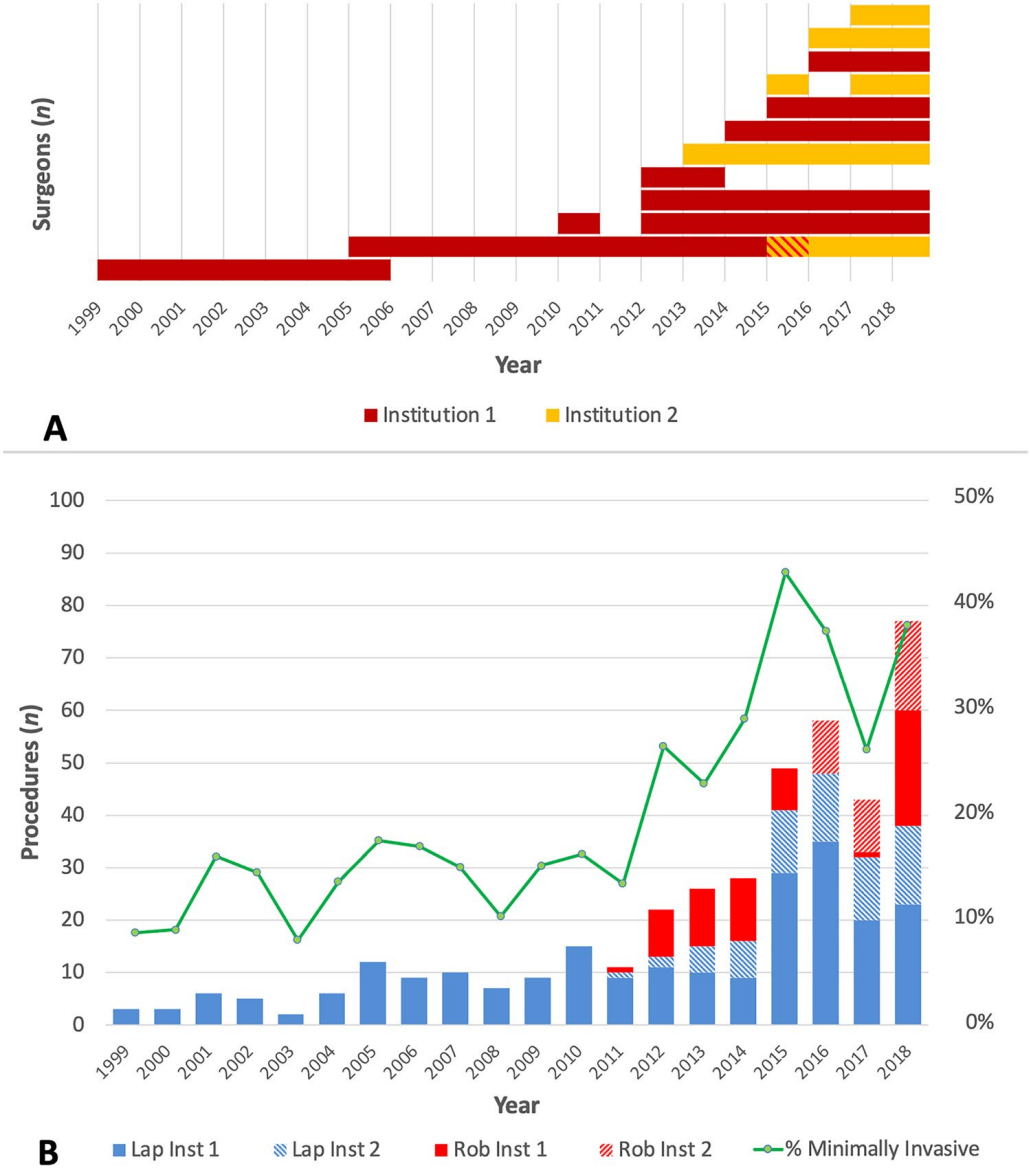
**A** Involvement of main surgeons over the years. **B** Number of laparoscopic and robotic distal pancreatectomy per institution and percentage of MIDP on the total of distal pancreatectomies per year, 1999 to 2018

During the study period, 401 patients were selected for MIDP (LDP *n* = 300, RDP *n* = 101) and a total of 12 surgeons performed the procedures. As shown in Fig. 1, main surgeons' involvement and the number of procedures increased exponentially from 2012 onwards. Particularly, during the early phase, the annual number of procedures performed overall in the two centres was less than 30, and the number of main surgeons involved never exceeded two. Instead, the annual volume was consistently higher than 40 cases/year during the recent phase, and the number of main surgeons involved varied between 7 and 10. Likewise, the percentage of MIDPs to the total number of distal pancreatectomies has increased over the years.

Patient demographics and pathologic variables are shown in Table 1. Cystic neoplasms were the most common surgical indication of the series (*n* = 152, 38%), followed by pancreatic neuroendocrine tumour in 139 (35%), and pancreatic ductal adenocarcinoma in 45 patients (11%). By stratifying patients by period, a proper case selection was evident during the early phase. A significant difference was observed in age, ASA score, and previous abdominal surgery (all *p* < 0.05). The number of patients affected by PDAC (15 vs. 30, *p* = 0.001), and the number of harvested lymph nodes (10 vs. 16, *p* < 0.001), increased during this time.

**Table 1:** Demographics and pathologic variables

	Overall	Early phase	Recent phase	*p*
*N*	401	201	200	
Years included	1999–2018	1999–2014	2015–2018	
Demographics				
Sex (Female)	277 (69%)	151 (75%)	126 (63%)	0.009*
Age, yr	52 (40–64)	49 (38–62)	55 (43–65)	**0.026**^**†**^
BMI, kg/m^2^	24 (21–27)	23.3 (20.3–26.9)	24.5 (21.6–27.5)	0.072^†^
BMI ≥ 25 kg/m^2^	155 (42%)	71 (38%)	84 (46%)	0.093*
Previous abdominal surgery	88 (22%)	20 (10%)	68 (34%)	< 0.001*
ASA				
1	65 (16%)	36 (18%)	29 (14%)	0.048*
2	313 (78%)	159 (79%)	154 (77%)	
3	23 (6%)	6 (3%)	17 (9%)	
Pathologic variables				
Cystic lesion	152 (38%)	95 (47%)	57 (28%)	< 0.001*
pNET	139 (35%)	64 (32%)	75 (37%)	
PDAC	45 (11%)	15 (7%)	30 (15%)	
SPT	42 (10%)	21 (11%)	21 (11%)	
Others	23 (6%)	6 (3%)	17 (9%)	
Size, mm	30 (20–41)	30 (20–45)	30 (20–40)	0.244^†^
Harvested lymph nodes	13 (6–21)	10 (4–19)	16 (8–24)	< **0.001**^**†**^

Bold values indicate statistical significance (*p* < 0.05)

All values presented as *n* (%), or median (IQR)

*BMI* Body Mass Index, *ASA* American Society of Anesthesiologists, *pNET* pancreatic Neuroendocrine Tumor, *PDAC* Pancreatic Ductal Adenocarcinoma, *SPT* Solid Pseudopapillary Tumor

*Pearson Chi-square

^†^Mann–Whitney U Test

### Intraoperative outcomes

Table 2 shows the intraoperative variables. The number of RDP increased significantly over time (20% vs 30%, *p* = 0.027). This data could justify the difference reported during the 2 periods in operative time (210 vs 265 min, *p* < 0.001). The spleen was preserved in 110 patients (27%), most frequently with the Kimura technique (n = 92, 83.6%). The percentage of SPDP of all MIDPs was reduced over time due to change in indications. The accuracy of surgical operation increased with time, as indicated by reductions of intraoperative blood transfusion (5% vs. 1%, *p* < 0.001). Furthermore, a decrease, but not significant, of the conversion rate was recorded (10% vs. 6%, *p* = 0.095). Use of the stapler device progressively increased over time (54% vs. 67%, *p* = 0.001).

**Table 2:** Intraoperative and postoperative outcomes

	Overall	Early phase	Recent phase	*p*
*N*	401	201	200	
Years included	1999–2018	1999–2014	2015–2018	
Intraoperative outcomes				
Conversion to open	31 (8%)	20 (10%)	11 (6%)	0.095
Approach: Lap	300 (75%)	160 (80%)	140 (70%)	0.027*
Rob	101 (25%)	41 (20%)	60 (30%)	
Spleen-preserving	110 (27%)	65 (32%)	45 (23%)	0.027*
Kimura	92 (84%)	54 (83%)	38 (84%)	0.849*
Warshaw	18 (16%)	11 (17%)	7 (16%)	
Operative time	235 (190–294)	210 (170–255)	265 (216–327)	** < 0.001**^**†**^
Transection technique				
Stapler	242 (60%)	109 (54%)	133 (67%)	0.001*
Ultrasonic dissector	144 (36%)	78 (39%)	66 (33%)	
Others	15 (4%)	14 (7%)	1 (1%)	
IO Blood transfusion	11 (3%)	9 (5%)	2 (1%)	< 0.001*
Postoperative outcomes				
Overall morbidity	194 (48%)	96 (47.8%)	98 (49%)	0.804*
Surgical morbidity	156 (39%)	71 (35.3%)	85 (42.5%)	0.141*
Clavien-Dindo ≥ 3	45 (11%)	22 (10.9%)	23 (11.5%)	0.860*
POPF	89 (22%)	40 (20%)	49 (25%)	0.268*
Grade B	78 (19%)	32 (16%)	46 (23%)	0.078*
Grade C	11 (3%)	8 (4%)	3 (2%)	
Biochemical leak	96 (24%)	43 (21%)	53 (27%)	0.231*
PPH	38 (10%)	20 (10%)	18 (9%)	0.745*
Blood transfusion	42 (11%)	19 (9%)	23 (12%)	0.755*
Intra-abdominal collection	98 (24%)	41 (20%)	57 (29%)	0.059*
Reoperation	36 (9%)	21 (10%)	15 (8%)	0.302*
Wound infection	3 (1%)	1 (0.5%)	2 (1%)	0.623^¶^
Medical morbidity	109 (27%)	58 (29%)	51 (26%)	0.450*
LoS	8 (6–11)	7 (6–10)	8 (7–12)	**0.002**^**†**^
Readmission (90-d)	42 (10%)	18 (9%)	24 (12%)	0.319*
Mortality (90-d)	0	0	0	–

Bold values indicate statistical significance (*p* < 0.05)

All values presented as n (%), or median (IQR)

*MI* Minimally Invasive, *Lap* Laparoscopy, *Rob* Robotic Surgery, *IO* intraoperative, *POPF* Post-Operative Pancreatic Fistula, *PPH* Post-Pancreatectomy Hemorrhage, *LoS* Length of Stay

*Pearson Chi-square

^†^Mann–Whitney U Test

^¶^ Fisher’s Exact Test

### Postoperative outcomes

Postoperative outcomes are described in Table 2. The overall surgical morbidity rate was 39%, without statistically significant difference between the periods (*p* = 0.141). Despite the increase in complexity of surgical cases, no differences were recorded in the rate of postoperative complication (all *p* > 0.05). However, a higher LoS was detected in the recent phase (7 vs. 8 days, *p* = 0.002). The stapler was the most used pancreatic transection technique, and the frequency of its use increased in the second period although a further analysis did not show differences in the POPF or PPH rate compared to the ultrasonic dissector (Table S1 in supplementary materials).

A subanalysis of the reasons for reoperation are shown in the supplementary materials (Table S2). Half of the cases were treated with a minimally invasive approach. Ninety-day mortality was nil.

### Learning curve

Preoperative variables and intra- and postoperative outcomes before and after completing the learning curve are shown in Table 3. For LPD, the operative time significantly decreased after the learning curve (240 vs 210 min, *p* < 0.001). Furthermore, a considerable reduction in postoperative complications and LoS were reported (*p* < 0.05). These events were not seen after RDP learning curve completion. However, during the learning curve, no patients underwent RDP for PDAC. Despite the increase in surgical difficulty, the intraoperative accuracy increased, with a lower intraoperative blood transfusion rate (*p* = 0.038).

**Table 3:** 17 LDP – 10 RDP

	First 17 LDP	Subsequent LDP	*P*	First 10 RDP	Subsequent RDP	*p*
*N*	138	162		45	56	
Demographics						
Sex (Female)	92 (67%)	113 (70%)	0.567*	11 (24%)	18 (32.1%)	0.395*
Age ≥ 65y	30 (22%)	33 (20%)	0.772*	10 (22%)	16 (28.6%)	0.468*
BMI ≥ 25 kg/m^2^	48 (38%)	66 (44%)	0.274*	17 (37%)	24 (43%)	0.525*
Prior abdominal surgery	21 (15%)	40 (25%)	0.114*	12 (27%)	15 (27%)	0.902*
ASA ≥ 3	13 (9%)	5 (3%)	0.021*	4 (9%)	1 (2%)	0.169^¶^
Intraoperative outcomes						
Conversion to open	15 (11%)	10 (6%)	0.142*	4 (9%)	2 (4%)	0.403^¶^
Spleen-preserving	39 (28%)	40 (25%)	0.484*	13 (29%)	18 (32%)	0.725*
Kimura	31 (80%)	33 (83%)	0.733*	11 (85%)	17 (94%)	0.849*
Warshaw	8 (20%)	7 (17%)		2 (15%)	1 (6%)	
Operative time	240 (200–296)	210 (163–255)	** < 0.001†**	274 (237–344)	280 (230–337)	0.730^†^
Transection technique						
Stapler	96 (70%)	107 (66%)	0.106*	14 (31%)	25 (44%)	0.381*
Ultrasonic dissector	33 (24%)	51 (32%)		30 (67%)	30 (54%)	
Others	9 (6%)	4 (2%)		1 (2%)	1 (2%)	
IO Blood transfusion	3 (2%)	4 (2%)	0.999¶	4 (9%)	0	**0.038**^¶^
Postoperative outcomes						
Overall morbidity	79 (57%)	65 (40%)	0.003*	23 (51%)	27 (48%)	0.772*
Surgical morbidity	61 (44%)	50 (31%)	0.017*	22 (49%)	23 (41%)	0.432*
Clavien-Dindo < 3	61 (44%)	51(31%)	0.023*	16 (36%)	21 (38%)	0.840*
Clavien-Dindo ≥ 3	18 (13%)	14 (9%)	0.218*	7 (16%)	6 (11%)	0.470*
POPF	31 (23%)	33 (20%)	0.659*	12 (27%)	13 (23%)	0.689*
Grade B	26 (19%)	29 (18%)	0.815*	11 (25%)	12 (21%)	0.922*
Grade C	5 (3%)	4 (2%)		1 (2%)	1 (2%)	
PPH	17 (12%)	14 (9%)	0.297*	4 (9%)	3 (5%)	0.697^¶^
Blood transfusion	14 (10%)	15 (9%)	0.739*	7 (16%)	6 (11%)	0.470*
Intra-abdominal collection	39 (28%)	28 (17%)	0.023*	16 (36%)	15 (27%)	0.342*
Reoperation	15 (11%)	12 (7%)	0.296*	5 (11%)	4 (7%)	0.507^¶^
LoS	8 (7–11)	7 (6–10)	**0.009†**	6 (8–12)	6 (8–13)	0.786^†^
Readmission (90-d)	12 (9%)	15 (9%)	0.865*	9 (20%)	6 (11%)	0.192*
90-d mortality	0	0	–	0	0	–
Pathologic variables						
PDAC	20 (14%)	17 (10%)	0.294*	0	8 (14%)	**0.008**^**¶**^
Harvested lymph nodes	14 (6–22)	16 (7–21)	0.492†	8 (3–17)	10 (3–19)	0.625^†^

Bold values indicate statistical significance (*p* < 0.05)

All values presented as n (%), or median (IQR)

^*^ Pearson Chi-square

^†^ Mann–Whitney U Test

^¶^ Fisher’s Exact Test

*LDP* Laparoscopic Distal Pancreatectomy, *RDP* Robotic Distal Pancreatectomy, *BMI* Body Mass Index, *ASA* American Society of Anesthesiologists, IO intraoperative, *POPF* Post-Operative Pancreatic Fistula, *PPH* Post-Pancreatectomy Hemorrhage, *LoS* Length of Stay, *PDAC* Pancreatic Ductal Adenocarcinoma

### Technical details

Table 4 shows data concerning preservation of the spleen. SPDP was associated with shorter operative time (195 vs 250 min, *p* < 0.001), and lower conversion rate (0% vs 11%, *p* < 0.001). Furthermore, the SPDP was correlated to a better postoperative course. Particularly, a decreased in morbidity (40% vs 52%, *p* = 0.039), POPF rate (12% vs 26%, *p* = 0.002), and LoS (7 vs 8, *p* = 0.010) were observed in the spleen-preserving group.

**Table 4:** Distal pancreatectomy with splenectomy vs Spleen-preserving

	DP-S	SP-DP	*p*	
*N*	291	110		
Intraoperative outcomes				
MI approach: Lap	221 (76%)	79 (72%)	0.396*	
Rob	70 (24%)	31 (28%)		
Conversion to open	31 (11%)	0	** < 0.001**^**¶**^	
Operative time	250 (210–304)	195 (153–240)	** < 0.001**^**†**^	
IO blood transfusion	9 (3%)	2 (2%)	0.735^¶^	
Postoperative outcomes				
Overall morbidity	150 (52%)	44 (40%)	0.039*	
Surgical morbidity	120 (41%)	36 (32.7%)	0.119*	
Clavien-Dindo ≥ 3	33 (11%)	12 (11%)	0.903*	
POPF	76 (26%)	13 (12%)	0.002*	
Grade B	67 (23%)	11 (10%)	0.025*	
Grade C	9 (3%)	2 (2%)		
Biochemical leak	68 (23%)	28 (25%)	0.662*	
PPH	28 (10%)	10 (9%)	0.871*	
Blood transfusion	37 (13%)	5 (5%)	**0.009**^**¶**^	
Intra-abdominal collection	73 (25%)	25 (23%)	0.624*	
Reoperation	26 (9%)	10 (9%)	0.961*	
LoS	8 (6–11)	7 (6–9)	**0.010**^**†**^	
Readmission (90-d)	28 (10%)	14 (13%)	0.365*	
90-d mortality	0	0	–	

Bold values indicate statistical significance (*p* < 0.05)

All values presented as n (%), or median (IQR)

*DP-S* Distal Pancreatectomy with Splenectomy, *SP-DP* Spleen-preserving Distal Pancreatectomy, *MI* Minimally Invasive, *Lap* Laparoscopy, *Rob* Robotic Surgery, *IO* intraoperative, *POPF* Post-Operative Pancreatic Fistula, *PPH* Post-Pancreatectomy Hemorrhage, *LoS* Length of Stay

*Pearson Chi-square

^†^Mann–Whitney U Test

^¶^Fisher’s Exact Test

### Follow-up

Ten patients (2.5%) lost to follow-up were not included in the long-term outcome analysis. Missing data regarding persistent thrombocytosis, vaccination adherence, and NODM were found (5%, 8%, and 3%, respectively). The median follow-up in the overall cohort (*n* = 391) was 52 months (IQR 28–85). Six patients crossed from SPDP to the DP-S group due to splenectomy during reoperation. Adherence to the post-splenectomy vaccination schedule was 89% in the first year of follow-up and dropped to 66% after five years. Two patients (0.5%) developed an overwhelming post-splenectomy infection (OPSI). One vaccinated patient developed pneumococcal meningitis one year after skipping revaccination but eventually survived. Even though vaccinated, the second patient died due to OPSI caused by *Capnocytophaga canimorsus* triggered by an underestimated domestic dog bite. Sixty-five patients (24%) reported having persistent thrombocytosis more than one year after DP-S, although no thrombotic events were reported.

The prevalence of preoperative DM in the whole cohort was 7.5% (29 patients) and of these 8 patients (2%) were already insulin-dependent. Preoperative DM was confirmed to be correlated with increase of age, male gender, and BMI ≥ 25 kg/m^2^ but was equally distributed according to the type of procedure (DP-S vs SPDP, 8.9% vs 4%, *p* = 0.115), the minimally invasive approach (LDP vs RDP, 8% vs 6.3%, *p* = 0.566), and the pathological class (PDAC vs other pathologies, 6.8% vs 7.7%, *p* = 0.999). Overall, 16.5% of patients developed type 3c NODM. Male gender (24% vs 13% of female gender, *p* = 0.016, Odds Ratio (OR) = 2, 95%CI [1.1–3.6]), increased age (≥ 65 y vs < 65 y, 27% vs 14%, *p* = 0.005, OR = 2.4, 95%CI[1.3–4.4]), BMI ≥ 25 kg/m^2^ (24% vs 8% of BMI < 25 kg/m^2^, *p* < 0.001, OR = 4.2, 95% CI [2.2–7.9]), DP-S (19% vs 10% of SPDP, *p* = 0.030, OR = 2.2, 95% CI [1.1–4.5]), and PDAC (33% vs 14% of other pathologies, *p* = 0.004, OR = 2.8 95% CI [1.4–5.9]) were associated with NODM.

## PDAC

**Fig. 2:**
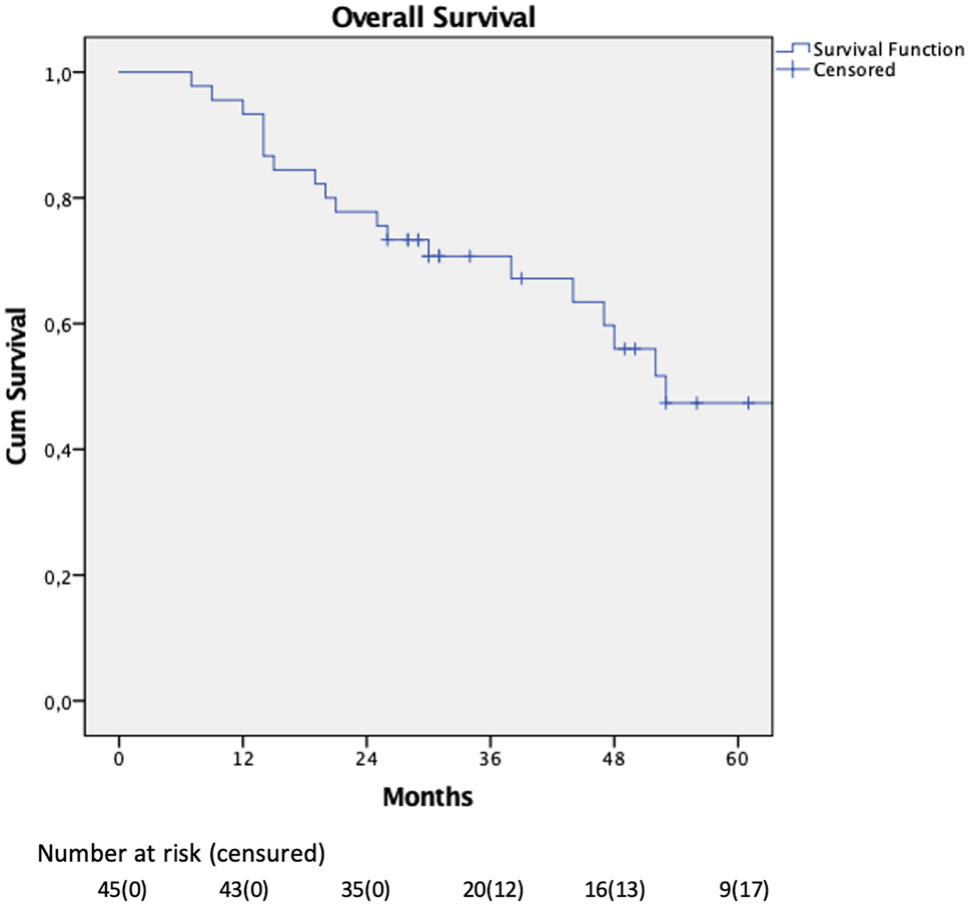
Overall survival in patients undergoing minimally invasive distal pancreatectomy for PDAC

Tables 5 and S3 (in supplementary material) summarise the specific results of the subgroup of MIDP performed for PDAC (n = 45). Notably 93% of specimen were R-zero. After a median follow-up of 31 months (IQR 25.5–53), the 1- and 3-year survival rates were 96% and 71%, respectively (Fig. 2). The estimated median survival time was 53 months (95% CI [29–77]). Among the 32 patients (71%) who developed recurrence, in 3 patients (7%) the first site involved was the abdominal wall at the level of trocar incisions. A fourth patient who underwent thoracoscopic resection of a single pulmonary metastasis developed recurrence at the thoracic trocar site.

**Table 5:** PDAC: pathological variables and follow-up

	N (%) or Median (IQR)
*n*	45
Pathologic variables	
Dimension, mm	30 (20–35)
R0	42 (93%)
Perineural invasion	41 (91%)
N1	29 (64%)
Follow-up	
Median follow up (IQR), months	31 (25.5–53)
1y survival	96%
3y survival	71%
Recurrence	32 (71%)
Trocar-site	3 (7%) + 1 thoracic trocar

## Discussion

The present analysis of 20 years of experience in MIDP collected 401 cases performed at two high-volume centres that adopted the same surgical techniques. Three main considerations can be made based on the results presented:

- Twelve surgeons were trained with increasing complexity of resection technique while maintaining an acceptable morbidity rate. Patient selection was progressively less strict for age and ASA class. In parallel, the surgical complexity increased due to more MIDP for PDAC and inclusion of patients with previous abdominal surgery.
- Forty-five PDAC were resected with 93% R-zero and 53 months of median survival.
- Spleen preserving distal pancreatectomies had a significantly lower complication rate when compared to DP with splenectomy.

Regarding the learning curve, several authors have addressed this issue for LDP [16, 32, 35–40]. The lack of a standardised training program for LDP makes the results of these studies difficult to compare [14]. For this reason, studies that explore the learning curve of MIDP need to be interpreted in the context of the case volume reported, number of main surgeons involved, and outcomes assessed. For example, the operative time, which was the most commonly used metric to estimate surgical proficiency, did not always decrease with the progression of cases in the published series [16, 35]. This result was consistent with the present series considering all the operators involved. Factors that can influence operative time are related to the surgical team (experience and skill of the first operator, assistant surgeons, and the scrub nursing staff), the patient (characteristics of the disease, sex, BMI, previous surgery, and frailty), and other contingent situations. Therefore, it is not surprising that in the literature the number of LDPs required to be completed to reduce operative time ranged from 10 to 80 cases [32, 37–40]. Furthermore, only Ricci et al. [32] analyzed a single surgeon series reporting a benefit following the completion of 17 cases. The present analysis divided the procedures performed by each operator before and after this threshold to overcome the bias deriving from individual skill level. The operative time reduction occurred in parallel with a drop of intra-abdominal collection, complications, and LoS. Notably, the extra-morbidity of the first cases was mainly represented by minor complications (Clavien-Dindo < 3). Although it is crucial to verify the safety of a surgical technique's learning process, postoperative outcomes are rarely used as a metric for evaluating the learning curve completion. In the context of LDP, only de Rooij et al.[35] reported an increased rate of major postoperative complications in the first 30 cases.

Like LDP, studies focusing on learning curve of RDP used operative time as the primary metric, and secondarily the estimated blood loss and readmission rate [14]. Again, the results had significant variability in the reported benefits after completing 5 to 40 cases [33, 41–43]. Among these, both Takahashi et al. [41] and Napoli et al. [33] analyzed RDPs performed by a single surgeon, identifying respectively 5 and 10 procedures as threshold values for improving operative time. In both series, as in the one described in the present study, the RDP learning curve started after acquiring adequate skills in LDP. Except for the decrease in intraoperative transfusions, the present study failed to identify improvements in operative time or other perioperative outcomes after 5 and 10 RDPs. At least two reasons may have contributed to this result. First, in both the cited studies, the authors reported an extensive and concomitant experience in robotic Whipple that likely contributed to shortening RDP's learning curve. In contrast, the two institutions in this study began to perform robotic resections of the pancreatic head only after the present study period. Second, the availability to access the robotic platform was limited for the study period's entire duration. For example, there was an 18-month period when the robotic platform was unavailable in one of the two institutions, thus lengthening the learning process.

Regarding the comparison of postoperative outcomes in spleen preservation and splenectomy, SPDP showed shorter operative time compared with DP-S, less morbidity, less need for blood transfusions, shorter LoS, and, most importantly, lower POPF rates. Nevertheless, several considerations need to be made when interpreting these results. First of all, SPDP constituted a selected group of patients affected exclusively by benign or low-grade malignant tumours. Secondly, an intention-to-treat analysis was not performed, and only successful procedures were included in the SPDP group as underscored by the absence of conversions. Although these results were in line with recent meta-analyses reporting that SPDP is associated with a lower incidence of infectious complications and POPF, shorter operative time, and less estimated blood losses [44–46], a large British propensity score-matched study, including distal pancreatectomy for benign or low-grade malignant tumours, showed no differences in postoperative morbidity [47]. There may be different interpretations of the favourable outcomes in SPDP reported here. It is reasonable to assume that the clinical procedure’s burden is different and that the surgical stress is reduced when the spleen is preserved. Furthermore, the increased incidence of POPF in DP-S could be attributable to the reduced vascularisation of the stump even if, at the moment, specific studies are lacking.

A further objective of the present analysis was to evaluate the long-term sequelae of the splenectomy. Postoperative vaccination is the basis for managing patients undergoing splenectomy [48]. However, multiple studies have documented poor adherence to post-splenectomy preventive measures [49, 50], which was consistent with the present findings. Vaccination protocol’s adherence was reduced from 89.1% to 66.2% over 5 years after the splenectomy. OPSI occurred with the same incidence (two cases, 0.5%) and mortality (one out of two cases) reported by current literature [51]. Concerning endocrine function, NODM incidence was 16.5%, confirming literature data [52]. Factors associated with NODM were male gender, BMI greater than 25, splenectomy, and PDAC, as already reported [52, 53]. Of note is the observation that one patient out of four had persistent piastrinosis after the splenectomy, leading to the necessity of cardioaspirin to prevent thrombosis.

The issue of pancreatic stump management after distal resection has been addressed by many clinical studies without a conclusive result. In the present series as in a recent randomized trial [54], there was no difference between stapler and ultrasonic dissector regarding the POPF or PPH rate.

The last issue examined in this analysis concerned a subgroup of patients with PDAC. The number of lymph nodes harvested was adequate and the rate of-R zero was high (93%) with a median tumour size of 3 cm. These remarkable results on one hand are related to a selection bias: PDAC with clear splenic artery and vein infiltration less than 5 mm from celiac trunk and porto-mesenteric junction were not approached with minimally invasive techniques. On the other hand, adequate pathological results encourage the choice of a minimally invasive approach in those selected patients. As a result of this accurate selection, a satisfactory long-term survival was observed (expected median survival of 53 months) although a relatively high rate of trocar site recurrence (7%) was reported. Some retrospective studies have shown an oncological advantage of LDP [55] and RDP [56] over the open approach. This survival benefit was attributed to lower blood transfusion and postoperative morbidity, as well as to an increased likelihood to receive adjuvant chemotherapy. Unbiased results are expected from the DIPLOMA trial (ISRCTN44897265) in this regard. Few reports have been published regarding trocar site recurrence after minimally invasive surgery for PDAC [57–59]. In colorectal surgery, the first reports of trocar site recurrences after laparoscopic surgery in the 1990s reported an incidence of up to 20% [60]. This alarming fact undermined the credibility of the laparoscopic approach for gastrointestinal malignancies. However, more recent large trials comparing laparoscopic and open colorectal surgery showed that the trocar site recurrence rate was about 1% which is not significantly different from abdominal wall recurrence after open surgery [61], but more studies focused on the oncological adequacy of minimally invasive surgery for PDAC are needed.

Several limitations apply to this study. First, the retrospective nature and the long study time should be taken into account when evaluating the results of this study. However, the main objective was to analyze the temporal evolution of indications, techniques, and the surgical team itself in MIDP. Second, the estimated blood loss was not evaluated in the analysis since it was not retrievable from first years of LDP medical records. Finally, the learning curve analysis of both LDP and RDP did not generate a cut-off for operative time reduction, so other data from the literature was borrowed.

Despite these limitations, this study demonstrates that in high-volume centres with a steadily increasing MIDP caseload, a less strict preoperative patient selection does not worsen the outcome. In academic centres with consolidated expertise and high case volume, it is possible to train young surgeons in MIDP while maintaining an acceptable morbidity rate. Moreover, whenever possible, SPDP should be preferred due to the excellent short- and long-term outcomes. Finally, with good patient selection, MIDP for PDAC offers adequate oncological outcomes in terms of pathological results and long-term survival.

## Supplementary Information

Below is the link to the electronic supplementary material.

Supplementary file1 (DOCX 35 kb)
