# Supplementary material for: 401 consecutive minimally invasive distal pancreatectomies: lessons learned from 20 years of experience
Source: Surg Endosc. 2022 Jan 31;36(9):7025–37. doi: 10.1007/s00464-021-08997-8 (PMC9402493; doi:10.1007/s00464-021-08997-8)
Supplement: Supplementary file 1 — Supplementary file1 (DOCX 35 kb) [file 464_2021_8997_MOESM1_ESM.docx]

**401 consecutive minimally invasive distal pancreatectomies: lessons learned from 20 years of experience**

**Supplementary materials**

| **TABLE S1.** Details on reoperations | | | |  |  |
| --- | --- | --- | --- | --- | --- |
|  | **N (%)** | | ***P*** |  |  |
| *n* | 36 (9%) | |  |  |  |
| Type of surgery | |  | | |  |
| Laparoscopic | 27 (9%) | | 0.978* |  |  |
| Robotic | 9 (8.9%) | |  |  |  |
|  |  | |  |  |  |
| DP-S | 26 (8.9%) | | 0.961* |  |  |
| SPDP | 10 (9.1%) | |  |  |  |
| Reason for reoperation |  | |  |  |  |
| Bleeding | 17 (47.2%) | |  |  |  |
| Abdominal abscess | 10 (27.7%) | |  |  |  |
| Splenic infarction | 3 (8.3%) | |  |  |  |
| Colonic perforation or ischemia | 3 (8.3%) | |  |  |  |
| Occlusion | 2 (5.6%) | |  |  |  |
| Severe pancreatitis | 1 (2.8%) | |  |  |  |
|  |  | |  |  |  |
| Reoperation approach |  | |  |  |  |
| Laparoscopic | 18 (50%) | |  |  |  |
| Open | 18 (50%) | |  |  |  |
| * Pearson Chi-square.  *DP-S* Distal Pancreatectomy with Splenectomy, *SP-DP* Spleen-preserving Distal Pancreatectomy | | | |  |  |

| **TABLE S2.** Pancreatic transection technique | | | | | | | |
| --- | --- | --- | --- | --- | --- | --- | --- |
|  | | **Stapler** | | **Ultrasonic dissector** | | ***P*** | |
| *n* | | 242 | | 144 | |  | |
| *Intraoperative outcomes* | |  | |  | |  | |
| MI Approach: Lap  Rob | | 203 (83.9%)  39 (16.1%) | | 84 (58.3%)  60 (41.7%) | | **<0.001*** | |
| Conversion to open | | 13 (5.4%) | | 6 (4.2%) | | 0.597 | |
| Spleen-preserving  Kimura  Warshaw | | 66 (27.3%)  52 (78.8%)  14 (21.2%) | | 41 (28.5%)  37 (90.2%)  4 (9.8%) | | 0.799*  0.124* | |
| Operative Time | | 230 (180–285) | | 250 (196–308) | | **0.015†** | |
| IO Blood transfusion | | 5 (2.3%) | | 3 (2.1%) | | 0.897* | |
| *Postoperative outcomes* | |  | |  | |  | |
| Overall morbidity | | 122 (51.8%) | | 64 (44.4%) | | 0.256* | |
| Surgical morbidity | | 95 (39.3%) | | 54 (37.5%) | | 0.732* | |
| POPF  Grade B  Grade C | | 51 (21.1%)  46 (19%)  5 (2.1%) | | 34 (23.6%)  28 (19.4%)  6 (4.2%) | | 0.561*  0.477* | |
| Biochemical Leak | | 52 (21.5%) | | 41 (28.5%) | | 0.121* | |
| PPH | | 24 (9.9%) | | 14 (9.7%) | | 0.950* | |
| Blood Transfusion | | 24 (10.7%) | | 14 (9.7%) | | 0.760* | |
| Abdominal Collection | | 54 (22.3%) | | 39 (27.1%) | | 0.289* | |
| Reoperation | | 22 (9.1%) | | 14 (9.7%) | | 0.837* | |
| Wound Infection | | 2 (0.8%) | | 1 (0.7%) | | 0.999¶ | |
| Medical morbidity | | 68 (28.1%) | | 37 (25.7%) | | 0.608* | |
| LOS | | 8 (6–11) | | 7 (6-10) | | 0.387† | |
| Readmission (90-d) | | 22 (8.7%) | | 20 (13.9%) | | 0.143* | |
| 90-d Mortality | | 0 | | 0 | | - | |
| All values presented as n (%), or median (IQR)  * Pearson Chi-square.  † Mann-Whitney U Test.  ¶ Fisher’s Exact Test.  *MI* Minimally Invasive, *Lap* Laparoscopy, *Rob* Robotic Surgery, *IO* intraoperative, *POPF* Post-Operative Pancreatic Fistula, *PPH* Post-Pancreatectomy Hemorrhage, *LOS* Length of Stay. | | | | | | | |

| **TABLE S3.** PDAC vs Distal pancreatectomy with splenectomy for other pathologies | | | | | | | | | | | | | |  |
| --- | --- | --- | --- | --- | --- | --- | --- | --- | --- | --- | --- | --- | --- | --- |
|  | | **PDAC** | | | | **Other pathology** | | | ***P*** | | | | |  |
| *N* | | | 45 | | | 246 | | |  | | | | | |
| *Demographics* | | |  | | |  | | |  | | | | | |
| Sex (Female) | | | 22 (49%) | | | 172 (70%) | | | **0.006*** | | | | | |
| Age ≥65y | | | 23 (51%) | | | 47 (19%) | | | **<0.001*** | | | | | |
| BMI ≥25kg/m^2^ | | | 15 (33%) | | | 95 (39%) | | | 0.979* | | | | | |
| Prior abdominal surgery | | | 18 (40%) | | | 53 (22%) | | | **0.008*** | | | | | |
| ASA ≥3 | | | 7 (16%) | | | 12 (5%) | | | **0.008*** | | | | | |
| *Intraoperative outcomes* | | |  | | |  | | |  | | | | | |
| Laparoscopy  Robot-assisted | | | 37 (82%)  8 (18%) | | | 184 (75%)  62 (25%) | | | 0.284* | | | | | |
| Operative time, *min* | | | 285 (230-330) | | | 245 (205-295) | | | **0.010†** | | | | | |
| Conversion to open | | | 9 (20%) | | | 22 (9%) | | | **0.027*** | | | | | |
| *Postoperative outcomes* | | |  | | |  | | |  | | | | | |
| Overall morbidity | | | 25 (56%) | | | 125 (51%) | | | 0.558* | | | | | |
| Surgical morbidity | | | 22 (49%) | | | 98 (40%) | | | 0.257* | | | | | |
| Clavien-Dindo ≥3 | | | 7 (16%) | | | 26 (11%) | | | 0.332* | | | | | |
| POPF  Grade B  Grade C | | | 17 (38%)  15 (33%)  2 (4%) | | | 59 (24%)  52 (21%)  7 (3%) | | | 0.053*  0.153* | | | | | |
| Chyle leak | | | 3 (7%) | | | 1 (0.5%) | | | **0.012¶** | | | | | |
| PPH | | | 4 (9%) | | | 24 (10%) | | | 0.999¶ | | | | | |
| Blood Transfusion | | | 9 (20%) | | | 28 (11%) | | | 0.120* | | | | | |
| Medical morbidity | | | 8 (18%) | | | 76 (31%) | | | 0.074* | | | | | |
| Wound Infection | | | 2 (4%) | | | 0 | | | **0.023¶** | | | | | |
| Reoperation | | | 6 (13%) | | | 20 (8%) | | | 0.261* | | | | | |
| Readmission (90-d) | | | 4 (9%) | | | 24 (10%) | | | 0.999¶ | | | | | |
| LOS | | | 8 (7-20) | | | 8 (6-11) | | | **0.044†** | | | | | |
| 90-d Mortality | | | 0 | | | 0 | | | - | | | | | |
| *Pathologic variables* | | |  | | |  | | |  | | | | | |
| Harvested lymph nodes | | | 26 (21–36) | | | 15 (9-21) | | | <**0.001†** | | | | | |
| All values presented as n (%), or median (IQR)  * Pearson Chi-square.  † Mann-Whitney U Test.  ¶ Fisher’s Exact Test.  *PDAC* Pancreatic Ductal Adenocarcinoma, *BMI* Body Mass Index, *ASA* American Society of Anesthesiologists, IO intraoperative, *POPF* Post-Operative Pancreatic Fistula, *PPH* Post-Pancreatectomy Hemorrhage, *LOS* Length of Stay. | | | | | | | | | | | | |  |  |
|  | |  | |  | | |  | | |  |  |  |  |  |
